# Posttransplant B cell Development and Function in Patients with B cell Positive SCID Caused by Pathogenic Variants in *IL2RG* and *JAK3*

**DOI:** 10.1007/s10875-026-02004-2

**Published:** 2026-03-21

**Authors:** Eva-Maria Jacobsen, Abdallah Khazaleh, Kerstin Felgentreff, Ingrid Furlan, Katharina Wustrau, Mehtap Sirin, Holger Cario, Benjamin Mayer, Ulrich Pannicke, Klaus Schwarz, Klaus-Michael Debatin, Wilhelm Friedrich, Ansgar S. Schulz, Manfred Hoenig

**Affiliations:** 1 Department of Pediatrics, University Medical Center Ulm, Eythstrasse 24, 89075 Ulm, Germany; 2 Institute for Transfusion Medicine, University of Ulm, Helmholtzstrasse 10, 89081 Ulm, Germany; 3 Institute for Clinical Transfusion Medicine and Immunogenetics Ulm, German Red Cross Transfusion Service Baden-Württemberg – Hessen, Helmholzstrasse 10, 89081 Ulm, Germany; 4 Institute for Epidemiology and Medical Biometry, Ulm University, Schwabstr. 13, 89075 Ulm, Germany

**Keywords:** B cell positive SCID, *IL2RG*, *JAK3*, HSC-transplantation, MZ-like B cells, B-lymphocyte reconstitution, Immunoglobulin substitution

## Abstract

Genetic defects in *IL2RG* or *JAK3* can cause the phenotype of severe combined immunodeficiency (SCID) with absent T- and non-functional B-lymphocytes (T-B+ SCID). B cell function and the need for immunoglobulin replacement therapy after hematopoietic stem cell transplantation (HSCT) depends on the engraftment of donor B-lymphocytes. In a retrospective study we describe B-lymphocyte reconstitution after HSCT with the aim to identify B cell subpopulations as an early predictor for the maturation and function of B cells after HSCT. All patients included underwent HSCT in a single institution between 1980 and 2017. First, we studied B cell maturation in cryopreserved blood samples of 12 patients with B+ SCID (IL2RG-deficiency) after haploidentical HSCT presenting with mixed B cell chimerism. Recipient and donor B cell subpopulations were identified by HLA-staining using flow cytometry. In a consecutive step we compared B cell subpopulations irrespective of chimerism between patients with or without post-transplant B cell function. Samples for this study were obtained between day + 90 to + 250 after HSCT from 25 post-transplant long-term survivors with B-positive SCID (9 with genetic variants in *JAK3*, 16 in *IL2RG)*, 9/25 were dependent and 16/25 independent of immunoglobulin (Ig) -substitution.

We demonstrate that a proportion of less than 2% of donor B cells can be sufficient for posttransplant B cell function and that a proportion of more than 4.7% of switched memory (IgM-) B cells in the memory B cell population (CD19 + CD27+) between days + 90 and + 250 after HSCT correlates with normal B cell function and independence from immunoglobulin substitution.

**Supplementary Information:**

The online version contains supplementary material available at 10.1007/s10875-026-02004-2.

## Introduction

Patients with B-lymphocyte positive SCID due to genetic variants in interleukin 2 receptor gamma (*IL2RG*) or janus kinase 3 (*JAK3*) need donor B cell engraftment to establish specific humoral immune functions after hematopoietic stem cell transplantation (HSCT). This is due to an intrinsic defect of recipient B cells to respond to IL-21 and to develop into mature class switched memory B cells and antibody producing plasma cells [1]. The common gamma chain (IL2RG, CD132) dimerizes with IL21 receptor alpha (IL21RA) and is an indispensable subunit of a functional receptor for IL-21 [2] with effective signal transduction via JAK-3. Primary therapeutic objective in patients with SCID is the establishment of a T cell system as this prevents from life threatening infections and allows for survival. The function of B-lymphocytes is dependent on T cell-help via CD40/CD40L and other coreceptor interactions in the germinal center as well as signaling via the IL-21 receptor in donor derived B cells [3, 4]. Currently, HSCT is the only established therapy to cure patients with B-positive SCID [5, 6]. Genetic correction of autologous stem cells by gene therapy is being evaluated in several studies [7, 8]. Before, during and early after these cellular therapies, patients are routinely treated with immunoglobulin replacement therapy. The decision to stop immunoglobulin (Ig)-substitution after successful HSCT is usually based on parameters such as the cessation of graft versus host disease (GvHD)-prophylaxis or -therapy, donor chimerism, quality of T cell reconstitution, immunoglobulin levels and as gold standard specific antibody responses to Tetanus toxoid or polysaccharide antigens of *H.influenzae* or *S.pneumoniae*.

This clinically important decision can be hampered by several aspects. Specific antigen titers and IgG-serum levels cannot be evaluated while patients are being treated with immunoglobulin replacement therapy. Testing for B cell chimerism can be complex as many patients present mixed chimerism after HSCT and therefore B cells have to be highly purified before short tandem repeat analysis (STR) or XY-fluorescence in-situ hybridization (FISH) to avoid any signal of potentially contaminating donor T cells. The minimal level of donor-B-lymphocyte chimerism sufficient to provide robust B cell function is not known. For an evidence based clinical decision Ig-substitution would have to be interrupted for a period of several months, during which patients are potentially at risk to acquire serious infections in case of a lack in B cell function.

The aim of this study was to investigate B cell development in patients with B cell positive SCID and mixed B cell chimerism after transplant and to identify informative parameters independent from Ig-substitution that correlate with the development of a functional B cell system as an early indicator for the cessation of immunoglobulin replacement therapy.

## Patients and Methods

Blood samples and clinical data were studied from patients treated between 1980 and 2017 at the University Medical Centre Ulm, Department of Pediatrics. Blood samples from pediatric healthy controls were collected and analysed between 2014 and 2015. Written informed consent was obtained from patients and/or their legal guardians. Research study protocols were approved by the local review board of Ulm University (reference/review number 20122017 and for pediatric healthy controls number 151/12) in accordance with the Declaration of Helsinki.

Flow cytometry studies were performed on mononuclear cells (MNCs) isolated from peripheral blood of patients taken at routine follow up appointments after HSCT. MNCs were either directly stained after isolation from fresh blood by density gradient cell separation (Biocoll Separating Solution, Biochrom AG) or obtained from frozen samples. Flow cytometry was performed on a NAVIOS 3L10C (Beckman Coulter) cytometer after washing and incubation with antibodies as indicated below. Clinical Data (patient age, genetic variants, transplant data, serum Ig-levels, need for Ig-replacement) were retrospectively collected from patient files.

### Chimerism Studies Based on HLA-Antigen Mismatches in Flow Cytometry

Samples from 12 long-term surviving patients affected by IL2RG deficiency and with mixed B cell chimerism after stem cell transplantation were analysed (cohort B). All patients were transplanted with grafts from haploidentical donors (mismatched related donor, MMRD). 9/12 received a myeloablative conditioning, 3/12 underwent HSCT without conditioning. The patients were analysed after a median follow up of 18.2 (1.5–34) years (see Table S1B). All patients were independent of Ig-replacement.

200 µl heparinized whole blood or 0,5-1 × 10E6 MNCs were incubated with unconjugated mouse human-leukocyte antigen (HLA)-antibodies against donor- and/or recipient HLA for 15 min at 4 °C followed by a washing step and incubation with FITC-conjugated secondary goat-anti-mouse IgM- and IgG-antibodies respectively for another 15 min at 4 °C. After two washing and erythrocyte-lysis steps, B cell subpopulations were further characterized in a second step with fluorescence conjugated antibodies. Results of B-cell subpopulations are shown as percentages of donor and recipient CD19 + B cells, respectively. Details on antibodies and fluorochromes are given in Table S2. In 6 out of 12 patients, antibodies, specific for recipient and donor HLA-antigens were available. In 4 patients, only anti-donor-type and in 2 patients, only anti-recipient-type HLA-antibodies could be tested. HLA positive and negative B cells were clearly distinguishable for gating. Positive and negative controls with HLA-typed cryopreserved buffy-coat MNCs were analysed in parallel with each experiment. Additional cell populations as monocytes, T and NK cells were characterized in the same experiment. As T cells are expected to originate completely from the donor in SCID-patients, these served as additional internal control. B cell phenotyping without HLA-staining led to equal results and distributions of subpopulations. If only one antibody was available, flow cytometric HLA results were confirmed once by an independent method (short tandem repeats analysis (STR) or XY-fluorescence-in-situ hybridization (FISH), data not shown) after separation of populations by magnetic beads (EasySep™ Cell Separation, STEMCELL technologies, Vancouver, Canada).

### B cell Immunotyping by Flow Cytometry

Samples from 25 patients with B-positive SCID treated with hematopoietic stem cell transplantation were included (cohort A, Table S1A). The selection of patients was based on the genotype and the availability of cryopreserved samples from the period of interest 90–250 days post HSCT. 7 patients of this cohort were also analysed for chimerism studies (annotated in Table S1). In 16 male patients, hemizygous variants in *IL2RG* were identified, in 9 patients, genetic variants affecting both alleles of *JAK3* were detected. At their most recent presentation, patients had a median follow up of 11.1 years (0.4–33.1) years after HSCT. Conditioning was based on variable regimen in 15/25 (60%) patients and 10/25 (40%) had been transplanted without conditioning (Table S1). Grafts were donated by mismatched family donors (MMFD) in 18/25, matched sibling donors (MSD) in 6/25 and a matched family donor (MFD) in 1/25 cases. Cryopreserved MNCs were thawed and used for flow cytometric B cell phenotyping using 0,5-1 × 10E6 cells for staining with fluorescence conjugated antibodies for 15 min at room temperature. B-cell subpopulations were analysed as percentages of CD19 + B cells. Definitions of B cell subpopulations and antibodies used for their identification by flow cytometry are given in Tables S3 and S2, respectively. A minimal cell population of 50 cells in the CD19 + CD27+ gate was considered necessary for the reliable analysis of subpopulations. Patient samples with less than 50 cells in this gate were excluded from the study.

### Definition of Patient Groups

According to the information taken from patient charts patients were either assigned to the immunoglobulin-dependent (IgDEP) or independent group (IgIND). All patients in the IgIND -group were demonstrated to have normal specific antibody responses after vaccination.

### Statistical Analysis

Statistical analysis was performed by using IBM SPSS Statistics 24 (Figs. 4, 5, S5, and S6) and GraphPad Prism 9.1.2. (Figs. S2 and S4), respectively. The Mann-Whitney U test was used to analyse differences between the IgIND and the IgDEP cohort (Fig. 4 and Fig S5). Differences between donor and recipient cell populations derived from the same patients were assessed using the Wilcoxon matched-pairs signed-rank test (Fig. S2), and correlations were evaluated using Spearman’s rank correlation coefficient (Fig. S4). In all analyses, a significance level below 0.05 was considered statistically significant. All analyses were exploratory in nature, and the resulting p-values do not support confirmatory inference. A predictive cut-off for immune reconstitution was defined by using receiver operating characteristics (ROC) analysis and determining the optimal cut-off value by maximizing the Youden index.

## Results

### HLA-Chimerism Analysis in Flow Cytometry: less than 2% of Donor B Cells can be sufficient for Posttransplant Specific Humoral Immune Function

**Fig. 1:**
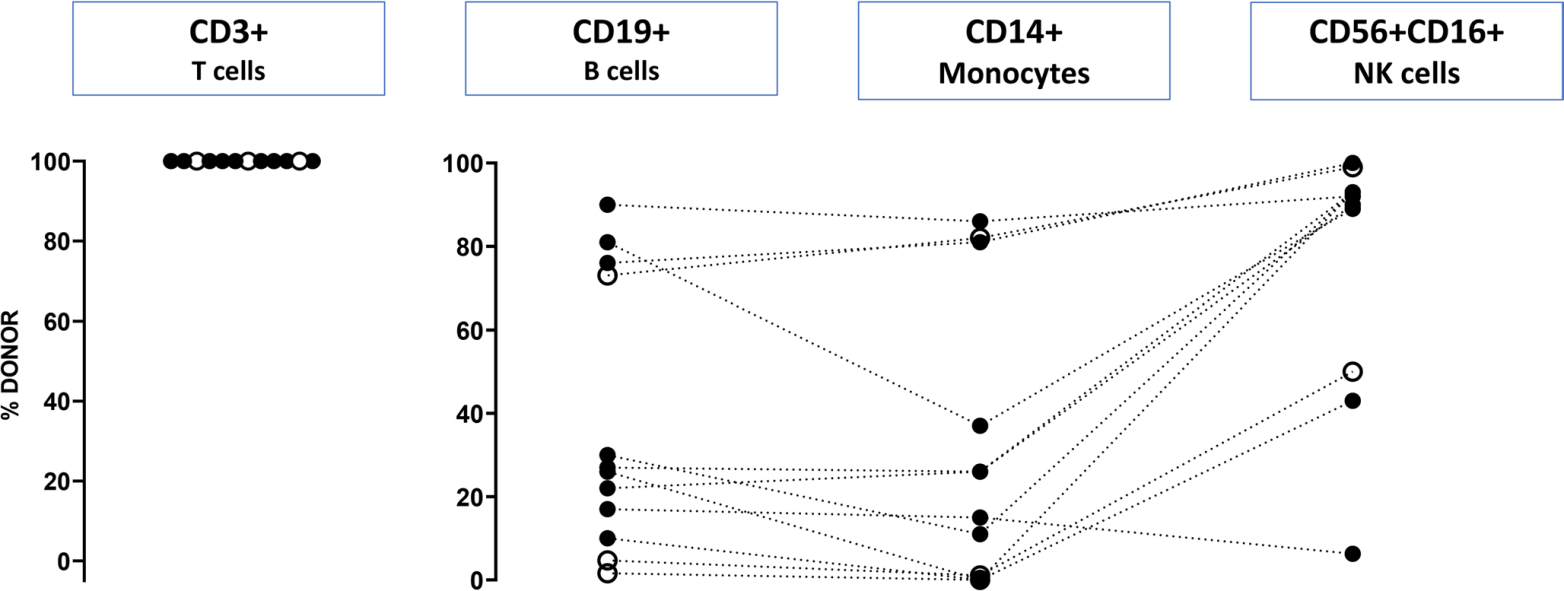
Donor chimerism in patients with IL2RG-deficiency after haploidentical HSCT. Chimerism analysis of B cells was performed by flow-cytometric analysis after staining with donor and/or recipient-specific HLA-Antibodies and CD19 (B cells), CD3 (T cells), CD56/16 (NK cells) and CD14 (monocytes) as lineage markers. Percentages of donor cells within the analysed cell population are shown. All patients are independent of immunoglobulin substitution. Patients who didn’t receive a conditioning regimen are shown with open circles. Dotted lines connect values of the same patients

 Samples of 12 patients (cohort B) were investigated in this part of the study, which originated from post-haplo-transplant survivors with B-lymphocyte positive SCID caused by genetic variants in *IL2RG*. All patients were off immunoglobulin replacement and had normal B cell function as determined by normal immunoglobulin serum levels for age and normal response to vaccinations. The majority of patients (9/12) received conditioning prior to HSCT while 3/12 did not (for details see Table S1B). As depicted in Fig. 1, T cells completely originated from the donor, whereas donor chimerism for B cells, monocytes and NK cells were mixed. B cell chimerism revealed to be highly variable between patients and a proportion of less than 2% of donor derived B cells was sufficient to allow for normal B cell function in this cohort. Monocyte donor chimerism was found at the detection limit for two patients. As expected, donor chimerism for NK cells is higher in comparison to monocytes in all but one patient as an indicator for a selective advantage of donor derived NK lymphocytes.

### B cell Maturation is Deficient in Autologous B Cells

**Fig. 2:**
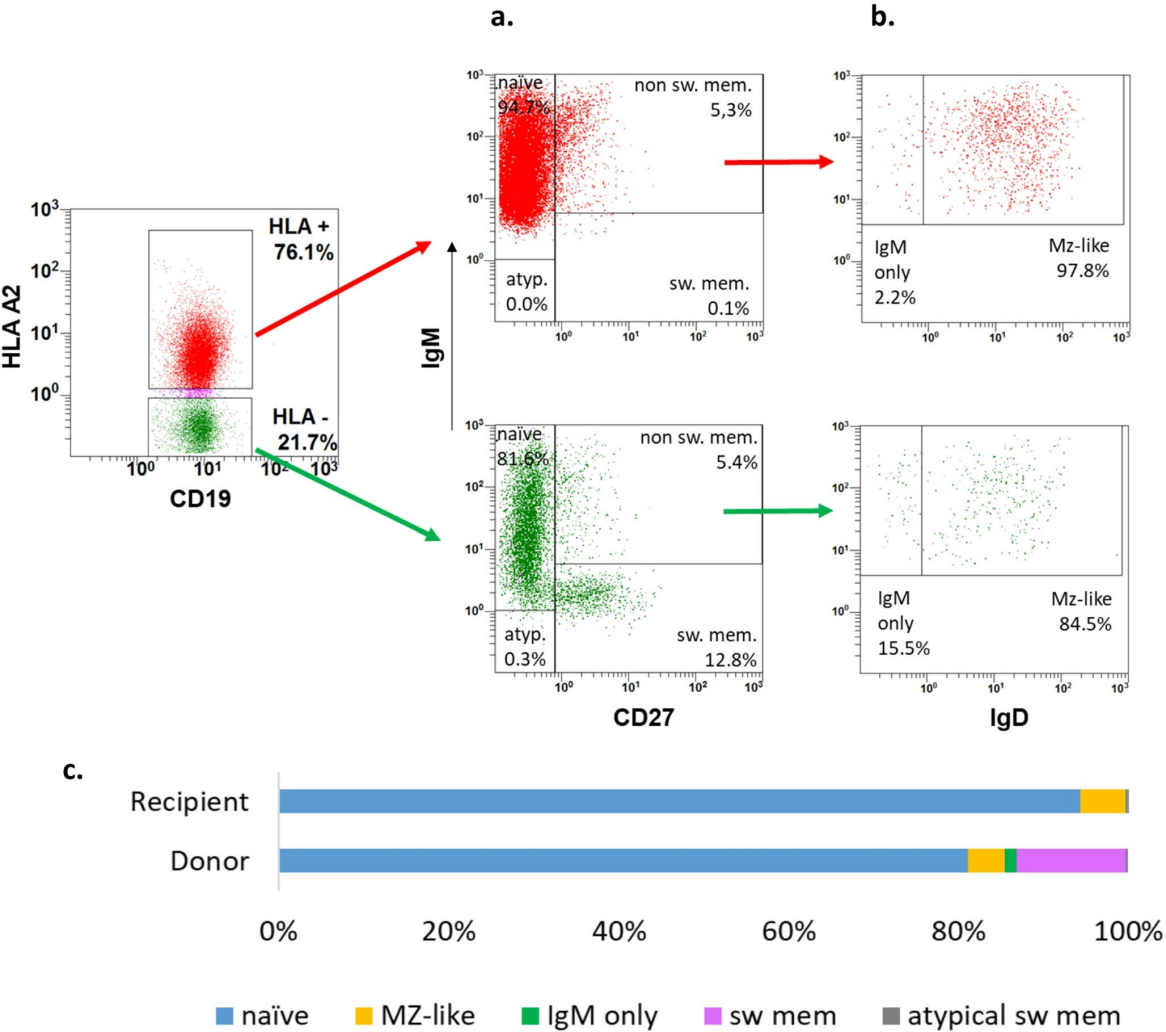
B cell maturation after HSCT is confined to donor B cells. Representative example of donor and recipient B cell subpopulations after HLA haploidentical SCT in a patient with IL2RG-deficiency. Peripheral blood MNCs (UPN 454, 14 years post SCT) were initially stained with HLA-A2 specific antibodies (recipient) followed by staining with anti-CD19, anti-CD27, anti-IgM and anti IgD. **a**. Only donor B cells (green: CD19+ and HLA-A2 neg.) are able to switch to IgM-CD27+CD19+ B cells while recipient B cells (red: CD19+ and HLA-A2 pos.) remain IgM+. **b**. Donor CD27+IgM+ (non switched) B cells show a substantial proportion of IgD- (IgM only) B cells while most of the IgM+CD27+ autologous B cells are IgD+. **c**. The distribution of B-cell subpopulations gated on CD19+ recipient and donor B cells, respectively, is shown. In donor B cells all stages of maturation, which are present in healthy controls, can be detected including switched memory B cells (red), IgM only memory B cells (green) and marginal-zone like B cells (orange). In recipient B cells mainly naïve B cells (blue) and a small proportion of marginal-zone like B cells are found. Definition of B cell subpopulations: IgD+CD27-: naive; IgM+CD27+: non switched memory; IgM-/CD27+: switched memory (sw mem); IgM-/CD27-: atypical memory (atypical sw mem); IgM+IgD+ (gate IgM+CD27+): Marginal Zone-like (MZ-like); IgM+IgD- (gate IgM+CD27+): IgM only. Fig. 2 B cell maturation after HSCT is confined to donor B cells

**Fig. 3:**
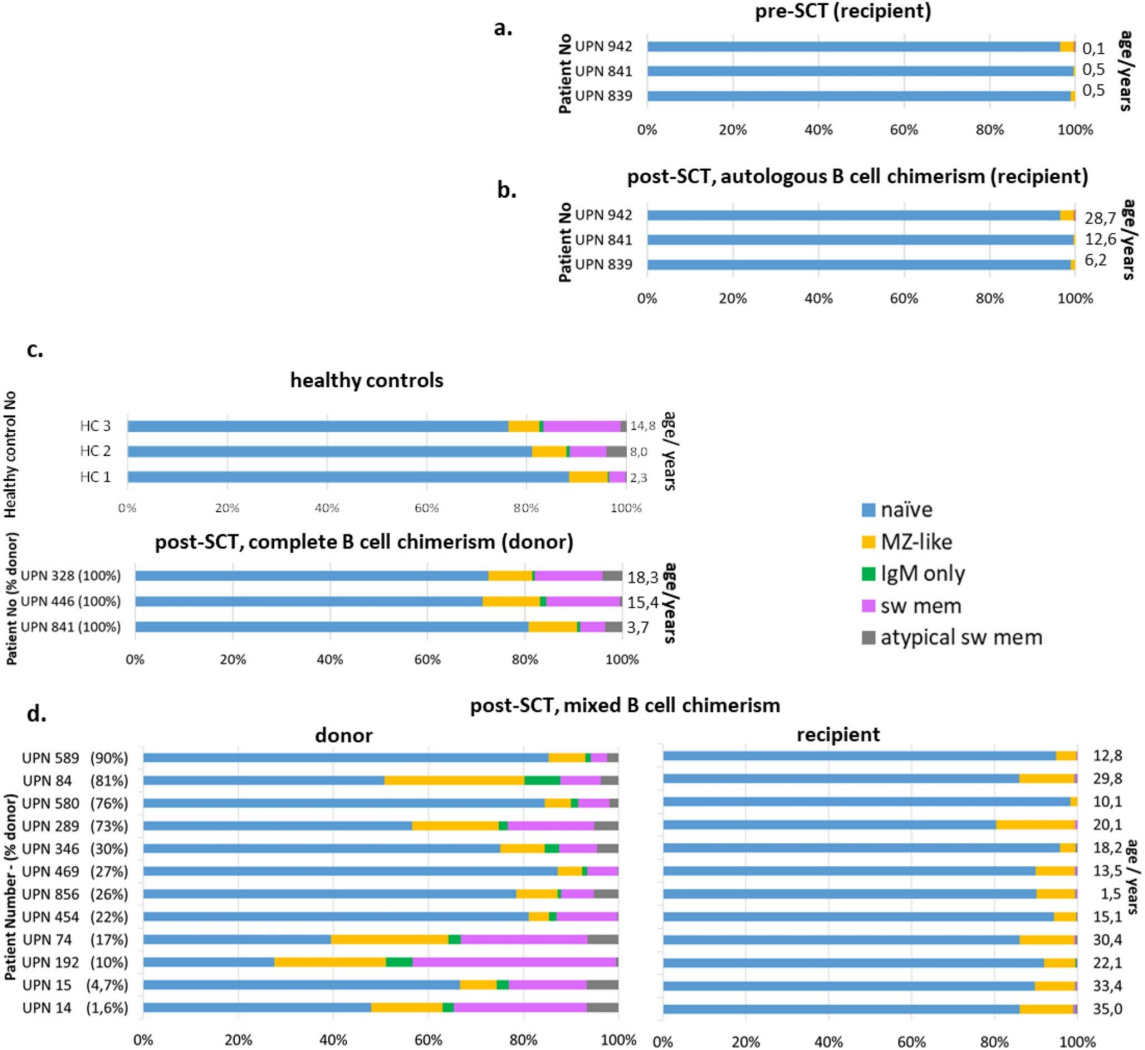
Distribution of B cell subpopulations in B cells of IL2RG-deficient patients pre- and post-SCT. The distribution of B-cell subpopulations (%) gated on CD19+ B cell (if indicated donor and recipient CD19+) is shown. B cells in patients pre SCT (**a**.) and in patients without donor B cells post SCT (**b**.) consisted predominantly of naïve B cells and a small proportion of Marginal-Zone like B cells while in pediatric healthy controls and patients with complete donor B cell chimerism (**c**.) all maturation stages including substantial proportions of switched memory B cells and IgM only B cells could be detected. **d**. The atypical distribution of B cell subpopulations in patients with mixed B cell chimerism with an accumulation of MZ-like B cells in the recipient B cell population (shown in detail in Fig 1 and Fig S1) was confirmed in 11 additional patients to patient UPN 454. B cells were analyzed with the same method of staining for donor (left) and recipient (right) specific HLA-alleles followed by staining for B cell subpopulations. Definition of B cell subpopulations: IgD+CD27-: naive; IgM+CD27+: non switched memory; IgM-/CD27+: switched memory (sw mem); IgM-/CD27-: atypical memory (atypical sw mem); IgM+IgD+ (gate IgM+CD27+): Marginal Zone-like (MZ-like); IgM+IgD- (gate IgM+CD27+): IgM only. The patients are listed according to their proportion of donor B cells (indicated in parentheses with the patient number)

As demonstrated for a representative sample in Fig. 2, HLA staining allows for donor and recipient specific analysis of B cell subpopulations in patients with post-transplant mixed B cell chimerism. If donor and recipient CD19-positive B cells are set to 100% respectively, the relative composition of the B cell compartments can be compared between donor and recipient (colored bars in Figs. 2 and 3) and between patients. This analysis exemplifies the remaining post-transplant maturational defect of autologous B cells despite the presence of donor T cells in all patients tested (Fig. 1) and the normal maturational potential of donor B cells.

Autologous switched memory B cells (CD19 + CD27+ IgM-) are hardly detectable whereas the majority (80–95%) of autologous B cells shows a naïve (CD19 + CD27-) phenotype with Marginal-Zone-like B cells as the dominant CD27 + population (Fig. 3 and Fig. S2). This latter pattern is shared by B-lymphocyte positive SCID patients pretransplant or posttransplant with autologous reconstitution (Fig. 3). This finding is in contrast to the experience in patients with other SCID entities such as IL7RA or Adenosine Deaminase (ADA) deficiency in whom autologous B cells develop maturational capacity as soon as a donor derived T cell system is established (Fig. S3).

In the donor B cell compartment a negative correlation (Spearman *r*= -0,678) between the percentage of donor B cells and the proportion of switched-memory B cells can be observed: the smaller the donor B cell compartment, the higher the percentage of switched-memory B cells (Fig. 3d and Fig. S4).

### Posttransplant Immunoglobulin Levels are of Limited Reliability as Indicators of B cell Function

**Fig. 4:**
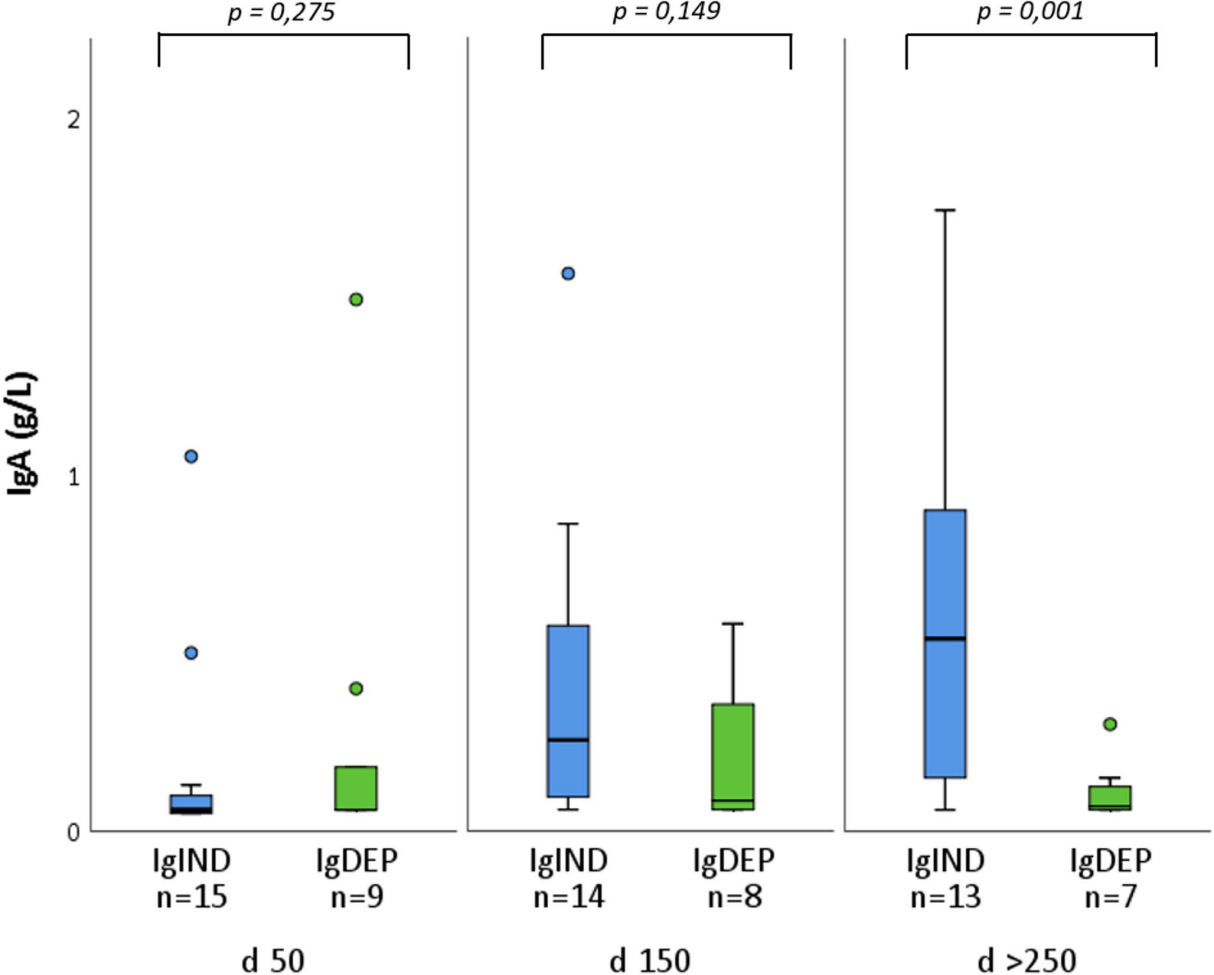
IgA-levels for patients with and without immunoglobulin substitution in different periods post-SCT. IgA-levels in patients (cohort A) at day 50, 150 and more than 250 days after hematopoietic stem cell transplantation (HSCT). IgA-levels in patients who do not need Ig-substitution (IgIND, blue colour) show a significantly higher IgA-level in the period at late time points > 250 days compared to patients who need Ig-substitution (IgDEP, green colour). In earlier periods, no difference can be detected. Boxes represent interquartile ranges (25.-75. percentile), lines within the boxes indicate median values while the whiskers extend to 1,5 times the interquartile range with individual points for outliers

To check for potential B cell function after HSCT, the clinical routine in most institutions would include the close observation of patient IgA- and IgM-levels as these are not contained in pharmaceutical immunoglobulin substitution products and therefore indicate a production by mature plasma cells. We were able to compare immunoglobulin levels at three periods post-HSCT (patients from cohort A): day 50, day 150 and between days 250 to 350. For IgA- and IgM levels, a significant difference between the IgIND and IgDEP group was only demonstrated after day 250 post transplant (Fig. 4, Fig. S5b).

### The Proportion of Switched Memory B cells in the CD27 + Pool Indicates the Extent of B cell Maturation and Correlates with Humoral Specific Immune Function

**Fig. 5:**
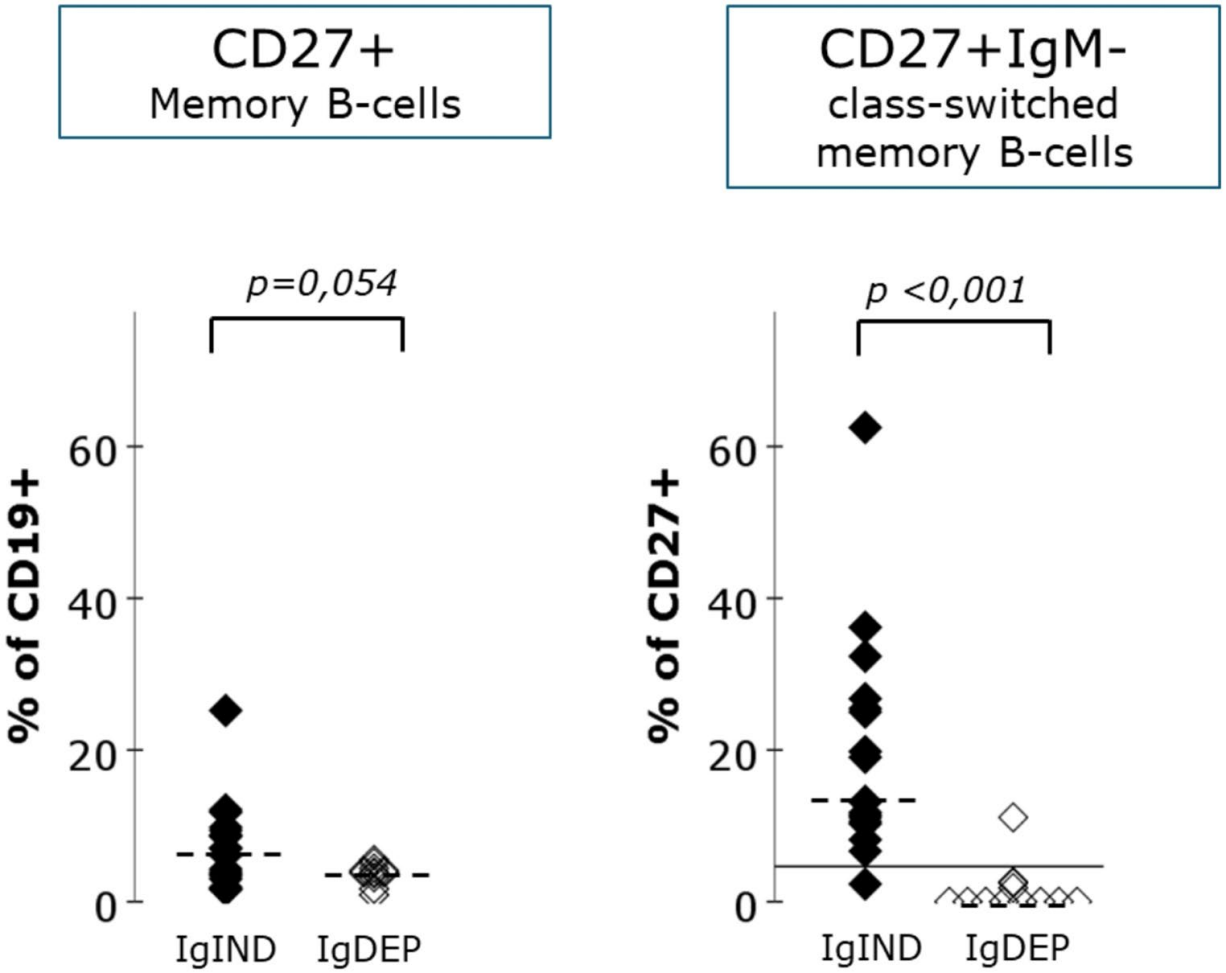
Comparison of proportions of B cell subpopulations for patients with and without the need for Ig-substitution. The proportion of memory B cells in the B cell compartment shows considerable overlap between the cohorts but the proportion of switched memory B cells in the memory B cell compartment is lower in the IgDEP group and shows an overlap for two patients only. The cutoff of 4.7% CD27+IgM- of CD27+ is indicated by a horizontal line. Median values are shown with the dotted horizontal line

In order to transfer the robust finding that autologous cells are not able to mature to switched-memory B cells as observed in patients after haploidentical transplantation (cohort B, Table S1B) to a more general and clinically more practicable approach we studied B cell subpopulations in cohort A irrespective of chimerism. Cohort A was -among other parameters- defined by the availability of cryopreserved samples taken from patients at routine follow up visits between 90 and 250 days post-HSCT. Patients in this cohort had been shown to have genetic variants in *IL2RG* and *JAK3* and were transplanted with grafts donated by mismatched family donors, matched unrelated donors and matched sibling donors (see Table S1). B cell phenotyping was performed without any discrimination between donor and recipient cells. Available data on chimerism from the same period after HSCT are given in Table S1. According to patient charts, 16 patients qualified for the IgIND and 9 patients for the IgDEP group with a median follow up of 11.7 years (0.4–33.1 years) for the IgIND group and 8.3 years (1-24.6 years) for the IgDEP group after HSCT respectively. As expected, patients in the IgDEP group show a lack of CD27-positive B cells in comparison to the IgIND group but this population is not completely absent. As demonstrated in cohort B (Figs. 2 and 3), autologous B cells can mature to a CD27 + stage, which is represented almost completely by Marginal-Zone like B cells. The clearest difference between autologous and donor B cell maturation in cohort B was found in the percentage of switched-memory B cells (CD19 + CD27+ IgM-) in the CD27 + B cell pool. This population was absent or considerably reduced in all patients except one in the IgDEP group (Fig. 5).

A ROC-analysis allowed to define a cut-off of 4.7% switched memory B cells in the CD27 + B cell pool to correctly assign a patient to the IgIND or IgDEP group with a specificity of 88.9% and a sensitivity of 93.8% (Fig S6).

The two patients of the IgIND and the IgDEP group, who were found below this threshold in the IgIND and above this threshold in the IgDEP group, were contacted and reevaluated. Patient 346 (Tab. S1), who was substituted with immunoglobulins but had a proportion of 11.1% of switched memory B cells in the CD27 + B cell pool, was contacted and paused Ig-substitution for 4 months during summertime. He was challenged with inactivated vaccines (Tetanus, Diphtheria, Haemophilus and Pneumococci) and checked for specific antibodies. These turned out positive and he is off IVIG since.

The proportion of switched memory B cells in patient 15 at the first analysis time point (d233 post HSCT) was still below the threshold of 4.7% in the CD27 + pool in spite of 14% donor B cells (Table S1). However, during 33 years of post HSCT follow up, he had no infectious history and was not substituted with immunoglobulins. His chimerism analysis at this late follow up time point (HLA-flow cytometry) revealed 4.7% of donor B cells with a proportion of 7.8% of switched memory B cells in the complete (donor and recipient) CD27 + B cell pool (not shown) and a proportion of 16% switched memory B cells in the donor B cells (Fig. 3d and S4a). IgG and IgM levels were found in the normal age-specific reference range but IgA was undetectable. Specific antibodies were positive for tetanus, pneumococci and rubella but negative for mumps and measles.

## Discussion

Without engraftment of donor derived B-lymphocytes, patients with SCID due to genetic variants in *IL2RG* and *JAK3* will remain without specific humoral immune function and thus dependent on immunoglobulin substitution [1]. The underlying intrinsic defect of autologous B cells has been previously reported for this patient group by White et al. [4] who demonstrated the inability of patient B cells, before and after HSCT, to perform a class switch and their inability to respond to IL-21 which was reported by Miggelbrink at al. [3]. With our approach to analyze CD19 + lymphocyte subpopulations and to label their donor or recipient origin by HLA-staining in flow cytometry, we provide further evidence for this primary defect in B cell maturation with an independent method. Beyond that, we can contribute an additional crucial clinical information: a very minor population of less than 2% of donor B cells in peripheral blood can be sufficient to allow for normal specific immune functions. The evaluation of immune function was based on a retrospective assessment of an uneventful infectious history, normal Ig levels and correct titers response to vaccinations in a cohort of patients with a median follow up of 14.3 (1.0-34.2) years. The formal weakness of this retrospective study turns to an advantage as this extensive follow up cannot be provided by a prospective approach and is a reliable clinical parameter to attribute patients in the cohort to the IgIND group.

We moreover observed the correlation of a low percentage of donor B cells with a high proportion of class-switched memory cells within this donor B cell population. This negative correlation could be interpreted as an attempt to compensate for the lack of functional B cells by a supranormal percentage of maturation to switched memory B cells within the donor derived and thus functional B cell population. This of course raises further questions on the stability of such a system of exploitation. From the clinical perspective, these patients do not show any phenotype of immunodeficiency but this does not exclude the possibility of abnormal findings regarding BCR variability, clonal expansion or cellular signs of senescence or exhaustion, which will have to be further elucidated.

The findings on B cells isolated from peripheral blood are not necessarily representative for the B cell system as a whole. Donor chimerism for B cell subpopulations in bone marrow and secondary lymphatic organs could vary considerably from those detected in CD19 positive cells in peripheral blood, but for obvious reasons this information will not be readily available. Even with these limitations, the information generated from blood samples remains essential for routine clinical decisions.

Autologous B cells after HSCT have a relevant proportion of 2.6 to 14% of CD27 + cells, which almost all turn out not to be memory B cells but MZ-like B cells as they stain positive for IgM and IgD. To our surprise this has not been recognized and reported in the literature until present. Marginal zone-like B cells and their function have not been well characterized in humans yet and their function in mice is described as an important component for a T cell independent innate first wave of IgM-antibody-reaction of the immune system to bind with low affinity to highly conserved motifs of infectious agents. Beyond this, MZ-like B cells have also been shown to be able to interact with follicular T-helper cells and undergo class switch reaction and somatic hypermutation as follicular B cells do [9]. In our studies we can clearly show, that CD19 + CD27+IgM + IgD+ cells can develop in the absence of signalling via the IL21-receptor but we strongly suspect that deep phenotyping and genetic studies (V(D)J-rearrangement and BCR-clonality) will be able to show major differences in this autologous B cell population in our cohort before HSCT (no T cell help, no IL-21 signalling), after HSCT (T cell help, no IL-21 signalling) and in comparison to donor derived MZ-like B cells (T cell help, normal IL-21 signalling) or samples from healthy controls.

We aimed to practically exploit these findings for clinical use to help with the decision to stop immunoglobulin substitution in post HSCT patients with B-positive SCID. This decision can be clinically challenging- especially for patients with mixed chimerism. The mere presence of B cells is not sufficient to deduce B cell function. As reviewed by van der Maas et al. [10], the reconstitution of non-switched Marginal-Zone like B cells and switched memory B cells proceeds slowly after HSCT and it can take up to two years or longer to reach age-matched normal levels. Gold standard and final proof for a functional B cell system is the detection of post-vaccination specific antibodies and Ig levels within the normal range in addition to an uneventful infectious history. For obvious reasons, IgG-levels and vaccination titers cannot be tested while patients are on Ig-substitution. IgM and IgA levels are used by many centers as indicators for normal specific humoral immune function [11, 12]. For our cohort, we could demonstrate that there is a significant difference between the IgIND and IgDEP groups for IgA and IgM-levels only at a period after 250 days posttransplant. IgA levels though remain with a major taint for clinical use as the absence of IgA in the age group tested here is within the normal range. The detection of donor derived B cells would be helpful but with the findings demonstrated above it becomes clear that a minor proportion donor derived B cells in the range of 2–5% cannot be reliably detected by other methods of chimerism analysis. Even after B cell enrichment or purification a percentage of less than 5% can be missed in STR analysis or XY-FISH. HLA-staining for flow cytometry is rarely established for routine chimerism analysis and is only feasible for patients transplanted from haploidentical donors. But with the experience of HLA-staining of B cell subpopulations we were able to clearly correlate the presence of CD19 + CD27+IgM- B cells in peripheral blood as indicator for B cell function. The quantification of this population irrespective of its chimerism in cryopreserved posttransplant patient samples allowed to statistically define a threshold of 4.7% CD27 + IgM- (% of CD27 + CD19+) with the highest specificity and sensitivity to retrospectively attribute a patient to the IgIND group.

The presence of this population of class-switched memory B cells was previously found to be informative for posttransplant B cell function for B cell positive SCID patients by Miggelbrink et al. [3] - we can confirm this finding in a larger cohort and add the clinically important quantification and definition of a threshold together with a diagnostic window as early as 90–250 days post HSCT. This additional information now allows the use of this parameter for clinical decision making and the cessation of post HSCT Ig-substitution. An earlier time point would not be informative as the help of follicular T cells is necessary to allow germinal center reactions to induce the class switch of B cells. The reconstitution of T cells after HSCT cannot be observed before 2–3 months after HSCT and can be delayed by GvHD, immunosuppressive drugs or infections. The potential impact of donor type or other parameters as stem cell source, GvHD or infections on the dynamics of B cell reconstitution would have to be investigated in a larger cohort and with longitudinal tests.

The clinical use of the quantification of CD19 + CD27+IgM- B cells is not confined to the cohort reported here but could be adapted to B cell positive SCID patients after autologous gene therapy or the growing group of patients after targeted B cell depleting therapies with monospecific (e.g. Rituximab) or bispecific (e.g. Blinatumomab) antibodies or CAR-T cells.

## Conclusion

In this retrospective analysis on patients with B cell positive SCID we could demonstrate by HLA-staining that a very minor percentage of less than 2% of donor B cells in peripheral blood correlates with normal immune function. Between day 90–250 in post-transplant follow up a proportion of > 4.7% of CD19 + CD27+IgM- within the CD27 + B cell pool is a sensitive and specific marker to indicate post HSCT B cell function in this cohort.

## Supplementary Information

Below is the link to the electronic supplementary material.

Supplementary Material 1

## Data Availability

The datasets generated during and/or analysed during the current study are available from the corresponding author on reasonable request.

## **Abbreviations**

ADA: Adenosine deaminase
BCR: B cell receptor
CD: Cluster of differentiation
CD40L: CD40 ligand
FISH: Fluorescence in situ hybridization
GvHD: Graft versus host disease
HLA: Human leucocyte antigen
HSCT: Hematopoetic stem cell transplantation
Ig: Immunoglobulin
IgIND: Independent of immunglobulin substitution
IgDEP: Dependent of immunglobulin substitution
IL: Interleukin
IL2RG: Interleukin 2 receptor gamma
IL7RA: Interleukin 7 receptor alpha
JAK3: Janus kinase 3
MNC: Mononuclear cells
MFD: Matched family donor
MMFD: Mismatched family donor
MSD: Matched sibling donor
MUD: Matched unrelated donor
MZ: Marginal zone
STR: Short tandem repeats
SCID: Severe combined immunodeficiency
