# Supplementary material for: Posttransplant B cell Development and Function in Patients with B cell Positive SCID Caused by Pathogenic Variants in IL2RG and JAK3
Source: J Clin Immunol. 2026 Mar 21;46(1):43. doi: 10.1007/s10875-026-02004-2 (PMC13102938; doi:10.1007/s10875-026-02004-2)
Supplement: Supplementary file 1 — Supplementary Material 1 [file 10875_2026_2004_MOESM1_ESM.docx]

**Supplemental Data**

**Posttransplant B cell development and function in patients with B cell positive SCID caused by pathogenic variants in *IL2RG* and *JAK3***

**Authors:** Eva-Maria Jacobsen^1,2^, Abdallah Khazaleh^1,2^, Kerstin Felgentreff^1^, Ingrid Furlan^1^, Katharina Wustrau^1^, Mehtap Sirin^1^, Holger Cario^1^, Benjamin Mayer^5^, Ulrich Pannicke^1,3^, Klaus Schwarz^3,4^, Klaus-Michael Debatin^1^, Wilhelm Friedrich^1^, Ansgar S. Schulz^1^, Manfred Hoenig^1^

**Institutions:**

^1^University Medical Center Ulm, Department of Pediatrics, Eythstrasse 24, 89075 Ulm, Germany;

^2^these authors have contributed equally

^3^ Institute for Transfusion Medicine, University of Ulm, Helmholzstrasse 10, 89081 Ulm, Germany

^4^Institute for Clinical Transfusion Medicine and Immunogenetics Ulm, German Red Cross Blood Transfusion Service Baden-Wuerttemberg-Hessen and University Hospital Ulm, Helmholtzstrass 10, 89081 Ulm, Germany

^5^Institute for Epidemiology and Medical Biometry, Ulm University, Schwabstr. 13, 89075 Ulm, Germany

**Corresponding Author:** Eva-Maria Jacobsen, University Medical Center Ulm, Department of Pediatrics, Eythstrasse 24, 89075 Ulm, Germany. Phone: 49 731 500 57262.

e-mail: [eva-maria.jacobsen@uniklinik-ulm.de](mailto:manfred.hoenig@uniklinik-ulm.de)

**Supplemental Tables**

**Table S1 Patient Data**

**Table S1A: Patient Data B cell Immunotyping**

| 1 | 2 | 3 | 4 | 5 | 6 | 7 | 8 | 9 | 10 | 11 | 12 | 13 | 14 | 15 | 16 | 17 |
| --- | --- | --- | --- | --- | --- | --- | --- | --- | --- | --- | --- | --- | --- | --- | --- | --- |
| **UPN** | **genetic variant** | **age at Tx [mo]** | **Donor type** | **conditioning** | **Regimen,**  units as deciphered  in the legend | **Sero**  **therapy** | **Serum IgM (g/l)** | **Serum IgA (g/l)** | **IVIG** | **CD27+/IgM-  >/= 4,65% of CD27+** | **Timepoint B-phenotype**  **on day+ after Tx** | **method of Chim Analysis** | ***Donor B cells/% / Non-T cells/%** | **Donor T cells/%** | **evidence of stem cell chimerism** | **absolute B-cell count (cells/µl)** |
| 15^b^ | *IL2RG* | 3 | MMFD | n |  |  | 0.72 | 0.18 | n | n | 233 | HLA | 14 | 100 | y (1) | - |
| 88 | *IL2RG* | 1 | MMFD | n |  |  | 3.46 | Below detection level | y | n | 127 | HLA | 0 | 100 | n (1) (2) | - |
| 246 | *JAK3* | 8 | MMFD | n |  |  | 1.9 | 0.57 | y | n | 141 | HLA | 0 | 100 | n (1) | 750 |
| 250 | *JAK3* | 6 | MMFD | n |  |  | 4.06 | 0.1 | y | n | 182 | HLA | 0 | 97 | n (1) | - |
| 289^b^ | *IL2RG* | 16 | MFD | n |  |  | 0.1 | 0.1 | n | y | 163 | HLA | 73 | 100 | y (1) | 971 |
| 346^b^ | *IL2RG* | 3 | MMFD | y | Bu8 |  | 1.85 | 0.62 | y | y | 140 | HLA | 30 | 100 | y (1) | 666 |
| 349 | *IL2RG* | 141 | MMFD | n |  |  | 2.53 | 2.2 | n | y | 100 | HLA | 100 | 100 | y (3) | 327 |
| 358 | *JAK3* | 5 | MMFD | y | Bu8 | ATG  20 | 0.1 | 0.1 | y | n | 103 | HLA | 1 | 100 | y (1) | 1617 |
| 368 | *JAK3* | 1 | MSD | n |  |  | 1.36 | 0.64 | n | y | 224 | - | n.a. | n.a. | n.a. | 2349 |
| 400 | *IL2RG* | 84 | MUD | y | Bu12.8 Cy200 | ATG  5 | 12.4 | 2.96 | n | y | 107 | STR | 100 | 100 | y (3) | 169 |
| 402 | *IL2RG* | 4 | MMFD | y | Bu8 |  | 0.33 | 0.06 | y | n | 154 | HLA | 15 | 100 | n (1) | 635 |
| 446 | *IL2RG* | 5 | MMFD | y | Bu12.8 Cy200 |  | 0.66 | 0.14 | n | y | 160 | HLA | 100 | 100 | y (3) | 93 |
| 454^b^ | *IL2RG* | 9 | MMFD | y | Bu12.8 Cy200 |  | 3.3 | 0.28 | n | y | 212 | HLA | 41 | 100 | y (1) | 981 |
| 469^b^ | *IL2RG* | 2 | MMFD | y | Bu12.8 Cy200 |  | 0.41 | 0.06 | n | y | 160 | HLA | 33 | 100 | y (1) | 1413 |
| 485 | *IL2RG* | 8 | MMFD | n |  |  | 0.21 | 0.06 | y | n | 120 | HLA | 2.5 | 100 | n (1) | 1141 |
| 506 | *JAK3* | 16 | MMFD | n |  |  | 0.38 | 0.21 | y | n | 185 | HLA | 7.3 | 100 | n (1) | 56 |
| 522 | *IL2RG* | 10 | MMFD | n |  |  | 0.32 | 0.08 | y | n | 107 | HLA | 0 | 100 | n (1) | 775 |
| 537 | *IL2RG* | 7 | MMFD | y | Bu12.8 Cy200 |  | 5.39 | 0.09 | n | y | 97 | HLA | 96 | 100 | y (1) | 89 |
| 558 | *JAK3* | 11 | MMFD | y | Bu16 Flu160 |  | 1.49 | 0.46 | n | y | 136 | HLA | 100 | 100 | y (1) | 1090 |
| 589^b^ | *IL2RG* | 6 | MMFD | y | Bu16 Cy120 Flu160 | ATG  10 | 0.06 | 0.06 | n | y | 239 | HLA | 90 | 100 | y (1) | 733 |
| 619 | *JAK3* | 7 | MSD | n |  |  | 0.21 | 0.25 | n | y | 126 | STR | Don > Rec | 100 | n (1) (2) | 1668 |
| 782 | *JAK3* | 2 | MSD | n |  |  | 0.05 | 0.14 | n | y | 162 | XY-FISH | 0 | 100 | n (1) | 1510 |
| 839 | *IL2RG* | 8 | MMFD | y |  | ATG  10 | 0.65 | 0.23 | n | y | 133 | XY-FISH | 96 | 100 | y (1) | 205 |
| 856^b^ | *IL2RG* | 6 | MMFD | y | Treo36 Flu160 | ATG  10 | 0.26 | 0.14 | n | y | 181 | HLA | 55 | 100 | n (1) | 718 |
| 875 | *JAK3* | 2 | MSD | n |  |  | 0.6 | 0.29 | n | y | 146 | STR | Rec >>> Don | 100 | n (1) | 880 |

**Table S1 B Patient Data Chimerism Analysis**

| 1 | 2 | 3 | 4 | 5 | 6 | 7 | 8 | 9 | 10 | 11 | 12 | 13 | 14 | 15 | 16 | 17 |
| --- | --- | --- | --- | --- | --- | --- | --- | --- | --- | --- | --- | --- | --- | --- | --- | --- |

| **UPN** | **gene** | **age at Tx [mo]** | **Donor type** | **conditioning** | **Regimen**  units as deciphered  in the legend | **Sero**  **therapy** | **Serum IgM (g/l)** | **Serum IgA (g/l)** | **IVIG** | **CD27+/IgM- >/= 4,65% of donor CD27+** | **Timepoint B-phenotype  year+ after Tx** | **method of Chim Analysis** | ***Donor B cells/%** | **Donor T cells/%** | **evidence of stem cell chimerism** | **absolute B-cell count (cells/µl)** |
| --- | --- | --- | --- | --- | --- | --- | --- | --- | --- | --- | --- | --- | --- | --- | --- | --- |
| 14 | *IL2RG* | 10 | MMFD | n |  |  | 4.2 | 1,34 | n | y | 34.2 | HLA | 1,6 | 99,7 | n (1) | 80 |
| 84 | *IL2RG* | 1 | MMFD | y | Cy120  Thio20 |  | 6.2 | ND | n | y | 29.7 | HLA | 81 | 100 | y (1) | 108 |
| 74 | *IL2RG* | 4 | MMFD | y | Bu16 Cy200 | ATG14 | 12.7 | 2,76 | n | y | 30.1 | HLA | 17 | 100 | y (1) | 311 |
| 192 | *IL2RG* | 9 | MMFD | y | Bu8 Cy100 |  | 6.3 | 1,73 | n | y | 21.2 | HLA | 10 | 100 | n (1) | 269 |
| 580 | *IL2RG* | 3 | MMFD | y | Bu16 Flu160 |  | 7.7 | 0,96 | n | y | 9.9 | HLA | 76 | 100 | y (1) | 547 |
| 15^b^ | *Il2RG* | 3 | MMFD | n |  |  | 11.4 | <0,05 | n | y | 33.1 | HLA | 4,7 | 100 | n (1) | 336 |
| 289^b^ | *IL2RG* | 16 | MMFD | n |  |  | 9.3 | 1,92 | n | y | 18.8 | HLA | 73 | 100 | y (1) | 235 |
| 346^b^ | *IL2RG* | 3 | MMFD | y | Bu8 |  | 7.6 | <0,05 | n | y | 17.9 | HLA | 21 | 100 | y (1) | 234 |
| 454^b^ | *IL2RG* | 9 | MMFD | y | Bu12.8 Cy200 |  | 8 | ND | n | y | 14.3 | HLA | 22 | 100 | y (1) | 531 |
| 469^b^ | *IL2RG* | 2 | MMFD | y | Bu12.8 Cy200 |  | 6.3 | 0,97 | n | y | 13.4 | HLA | 28 | 100 | y (1) | 471 |
| 589^b^ | *IL2RG* | 6 | MMFD | y | Bu16 Cy120 Flu160 |  | 7.7 | 0,81 | n | y | 12.3 | HLA | 90 | 100 | y (1) | 344 |
| 856^b^ | *IL2RG* | 6 | MMFD | y | Treo36 Flu160 |  | 3.4 | 0,26 | n | y | 1.0 | HLA | 26 | 100 | n (1) | 709 |

**Table S1.** Key information on the study participants. **S1 A:** study participants for B cell immunotyping in flow cytometry (referred to as cohort A). **S1 B:** study participants for chimerism studies based on HLA-antigen mismatches in flow cytometry (referred to as cohort B). Patients analysed for both purposes are indicated (^b^).

UPN 289 and 349 were retransplanted after graft failure, B cell phenotyping was performed after second BMT. For the conditioning regimen, medication and cumulative dose (Busulfan (Bu) mg/kg; Fludarabin (Flu) mg/m^2^; Cyclophosphamide (Cy) mg/kg; Treosulfan (Treo) g/m^2^; Antithymoglobulin (ATG) mg/kg and Thiotepa (Thio) mg/kg) is given. The time point of denoted chimerism-data was chosen closest to the sample date for B cell phenotyping (0-0.6 y). Of 4 patients, only chimerism results at later time points (UPN15: 6.3y UPN88: 12.7y UPN 246: 1.1y UPN782: 2.2y) were available. For chimerism studies based on HLA-antigen mismatches (table S1B), time points of chimerism-analyses and B cell phenotyping were identical. *Donor Chimerism of B cells is given, if HLA-chimerism analysis was performed, for other chimerism methods, donor chimerism of non-T cells is denoted. Evidence of stem cell chimerism was classified as positive (y) if at least one of the following 3 criteria was fulfilled: (1): monocytes or granulocytes: donor present in STR or > 5% donor in HLA/XY FISH analysis, (2): CD34+ stem cells: donor present in STR or > 5% donor in HLA/XY FISH analysis, (3): full blood: 100% donor. The timepoint of analysis after transplant is given in table S1A (column 12, days after HSCT) and S1B (column 12, years after HSCT). All patients were off immunosuppressants at the time when samples were taken. Abbreviations: [mo]: months; HLA: human leukocyte antigen / flow cytometric chimerism analysis by using anti-HLA antibodies; XY-FISH: chimerism analysis by fluorescence-in-situ hybridization of X and Y chromosomes; STR: chimerism analysis by short tandem repeat analysis; Rec: recipient; Don: donor; MMFD: mismatched family donor; MFD: matched family donor; MUD: matched unrelated donor; MSD: matched sibling donor.

**Table S2 Antibodies used for immunophenotyping**

| **Specificity** | **Fluorochrome** | **Clone** | **Isotype** | **Dilution** | **Catalog No** | **Company** |
| --- | --- | --- | --- | --- | --- | --- |
| CD3 | APC | UCHT1 | IgG1 mouse | 1:20 | IM2467 | Beckman Coulter,  Krefeld Germany |
| CD14 | FITC | TÜK4 | IgG2a mouse | 1:20 | F0844 | Dako/ Agilent, Santa  Clara, CA, USA |
| CD16 | ECD | 3G8 | IgG1 mouse | 1:50 | B49216 | Beckman Coulter,  Krefeld Germany |
| CD19 | APC-Alexa Fluor 700 | J3-119 | IgG1 mouse | 1:50 | B49212 | Beckman Coulter,  Krefeld Germany |
| CD27 | Horizon V450 | M-T271 | IgG1 mouse | 1:50 | 560448 | Becton Dickinson,  San José, CA, USA |
| CD45 | Krome-Orange | J.33 | IgG1 mouse | 1:50 | B36294 | Beckman Coulter,  Krefeld Germany |
| CD56 | ECD | N901 (NKH1) | IgG1 mouse | 1:20 | B49214 | Beckman Coulter,  Krefeld Germany |
| IgA | PE | polyclonal | F(ab`)2 goat | 1:500 | 2052-09 | Southern Biotech,  Birmingham, AL, USA |
| IgD | FITC | polyclonal | F(ab`)2 Rabbit | 1:20 | F0189 | Dako/ Agilent, Santa  Clara, CA, USA |
| IgD | PE-Cy7 | IA6-2 | IgG1 mouse | 1:50 | 348210 | Biolegend, San Diego,  CA, USA |
| IgG | PE-Cy7 | G18-145 | IgG1 mouse | 1:20 | 561298 | Becton Dickinson,  San José, CA, USA |
| IgM | Alexa Fluor 647 | polyclonal | F(ab`)2 Goat | 1:20 | 314510 | Jackson ImmunoResearch, Cambridgeshire, UK |
|  |  |  |  |  |  |  |
| **HLA-antibodies** |  |  |  |  |  |  |
| A1,11,26 | unconjugated | monoclonal | IgM mouse | 1:10 | 0544 HA | One Lambda INC,  Kittridge, CA, USA |
| A2 | unconjugated | monoclonal | IgM mouse | 1:4 | 0475 HA | One Lambda INC,  Kittridge, CA, USA |
| A3 | unconjugated | monoclonal | IgM mouse | 1:5 | 0170 HA | One Lambda INC,  Kittridge, CA, USA |
| A 11 (+/-A3) | unconjugated | monoclonal | IgG mouse | 1:4 | 0284 HA | One Lambda INC,  Kittridge, CA, USA |
| A23/24 | unconjugated | monoclonal | IgG2b mouse | 1:5 | 0041 HA | One Lambda INC,  Kittridge, CA, USA |
| A 29 | unconjugated | monoclonal | IgM mouse | 1:5 | 0334 HA | One Lambda INC,  Kittridge, CA, USA |
| A 30/31 | unconjugated | monoclonal | IgM mouse | 1:5 | 0273 HA | One Lambda INC,  Kittridge, CA, USA |
| B8 | unconjugated | monoclonal | IgM mouse | 1:5 | 0059 HA | One Lambda INC,  Kittridge, CA, USA |
| B8,14 | unconjugated | monoclonal | IgM mouse | 1:4 | 0332 HA | One Lambda INC,  Kittridge, CA, USA |
| B13,62,15 | unconjugated | monoclonal | IgG mouse | 1:10 | 0044 HA | One Lambda INC,  Kittridge, CA, USA |
|  |  |  |  |  |  |  |
| **secondary antibodies** |  |  |  |  |  |  |
| Goat anti Mouse IgG (H+L) | FITC | polyclonal | F(ab`)2 goat | 1:100 | 1032-02 | Southern Biotech,  Birmingham, AL, USA |
| Goat anti Mouse-IgM | FITC | polyclonal | F(ab`)2 goat | 1:100 | 1022-02 | Southern Biotech,  Birmingham, AL, USA |

**Table S3 Definition of B cell subpopulations**

| **Definition of B cell subpopulations (gated on CD19+ B cells)** | | | |
| --- | --- | --- | --- |
|  |  |  |  |
| naïve | CD27-IgD+(IgM+) | |  |
| memory | CD27+ |  |  |
| non-switched memory | CD27+IgM+ | |  |
| IgM-only | CD27+IgM+IgD- | |  |
| Marginal Zone-like | CD27+IgM+IgD+ | |  |
| class-switched memory | CD27+IgM- | |  |
| atypical memory | CD27-IgM- |  |  |

**Supplemental Figures**

**Supplemental Figure S1:** **Complementary HLA-staining**

**
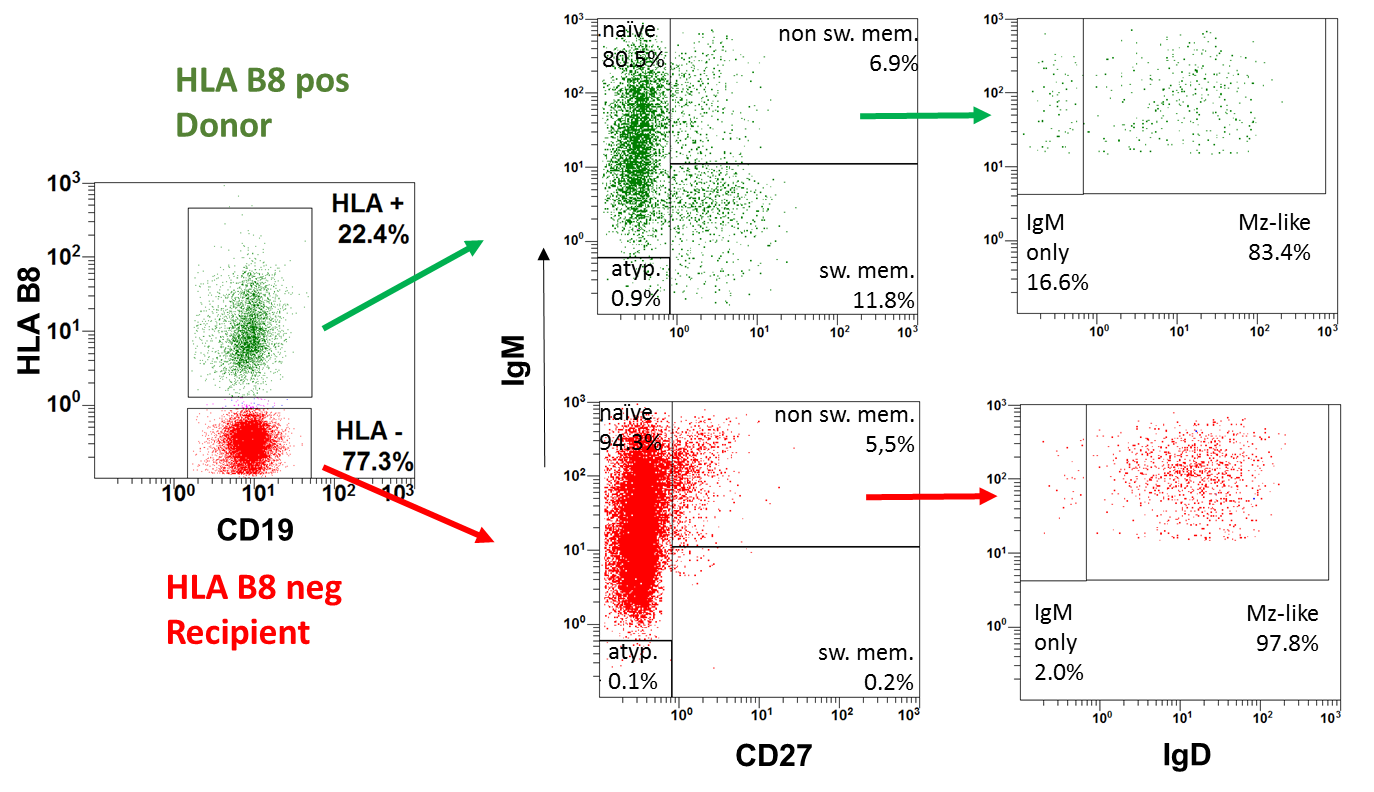
**

**Figure S1**. Staining with donor specific antibodies of the same sample obtained from patient UPN 454 confirms the results produced with a recipient specific antibody shown in Fig 2. This complementary staining excludes potential artefacts by false positive or false negative binding of HLA-specific antibodies. Patient peripheral blood MNC (14 years post SCT) were stained with HLA-B8 (donor). After staining with the HLA-specific antibody, MNCs were subsequently stained with anti-CD19, anti-CD27, anti-IgM and anti IgD. Donor B cells (green: CD19+ and HLA-B8 pos., upper row) are able to switch to IgM-CD27+CD19+ B cells while recipient B cells (red: CD19+ and HLA-B8 neg., lower row) are IgM+. Donor CD27+IgM+ (non switched mem) B cells show a substantial proportion of IgD- (IgM only) B cells while most of the IgM+CD27+ autologous B cells are IgD+.

Definition of B cell subpopulations: IgD+CD27-: naïve; IgM+CD27+: non switched memory; IgM-/CD27+: switched memory (sw mem); IgM+IgD+ (gate IgM+CD27+): Marginal Zone-like (MZ-like); IgM+IgD- (gate IgM+CD27+): IgM only

**Supplemental Figure S2: Distribution of donor and recipient B cell subpopulations**

A.

CD27-IgD+(IgM+)
**Naïve B cells**

CD27+IgM-
**Class-switched memory B cells**

CD27+
**Memory B cells**

CD27+IgM+
**Non-switched memory B cells**

B. C.

CD27+IgM-
**Class-switched memory B cells**

CD27+IgM+IgD-
**IgM only B cells**

CD27+IgM+IgD+
**Marginal Zone-like B cells**

**Figure S2 A.** **Distribution of donor and recipient B cell subpopulations of CD19+ B cells.** Distribution of naïve, CD27+, switched memory (CD27+/IgM-) and non switched memory (CD27+IgM+) B cells gated on donor (D, filled circles) and recipient (R, open circles) CD19+ B cells: switched memory B cells are only present in the donor-cell population while naïve, CD27+, and non switched MZ-like B cells are present in both recipient and donor B cells. **B. Distribution of donor and recipient B cell subpopulations of CD27+IgM+ B cells.** Proportion of IgM only B cells within the CD27+IgM+ population: in the donor cell population 5-25% of the CD27% were identified as IgM only and 75-94% as Marginal Zone like B cells, while the recipient CD27+ B cells only showed very low percentages (0-2,7%) of IgM only and >95% of Marginal Zone-like B cells. **C. Proportion of switched memory B cells within the CD27+ population:** in the donor-cell population 19-70% of the CD27% were IgM negative switched memory cells, while the recipient CD27+ B cells showed no or very low proportions of switched memory B cells. The Wilcoxon matched-pairs signed rank test was used for comparing median black values (black lines) between donor and recipient populations. p-values and statistical significance are indicated. ns: non significant.

**Supplemental Figure S3: Normal distribution of autologous B cell subpopulations in patients with ADA-deficiency or IL7R-Deficiency after HLA-haploidentical transplantation.**

**
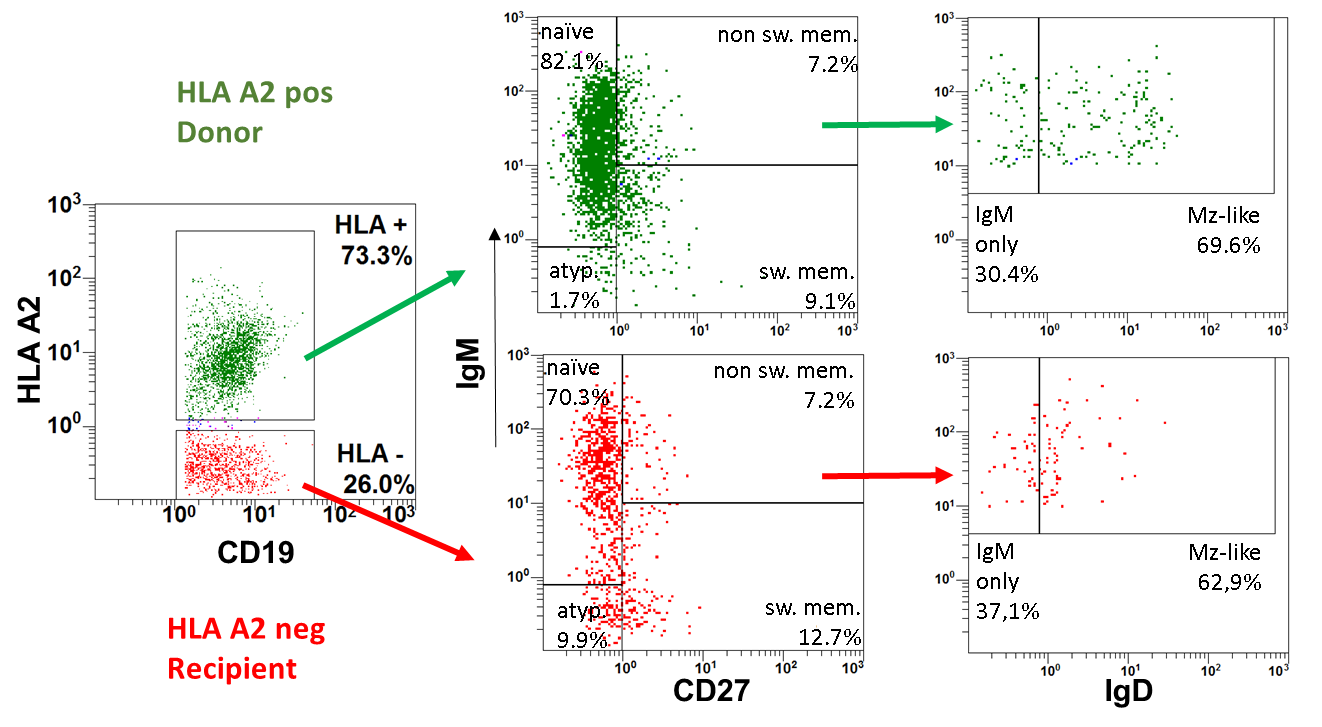
A.**

**B.**


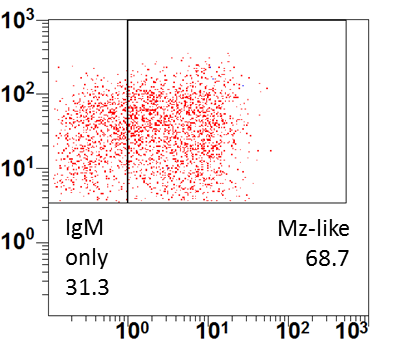

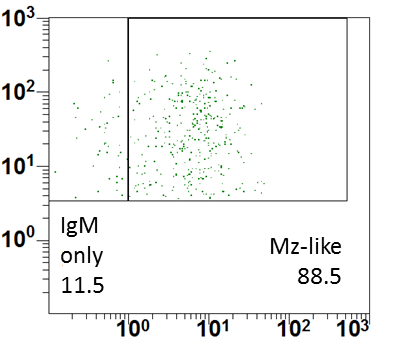

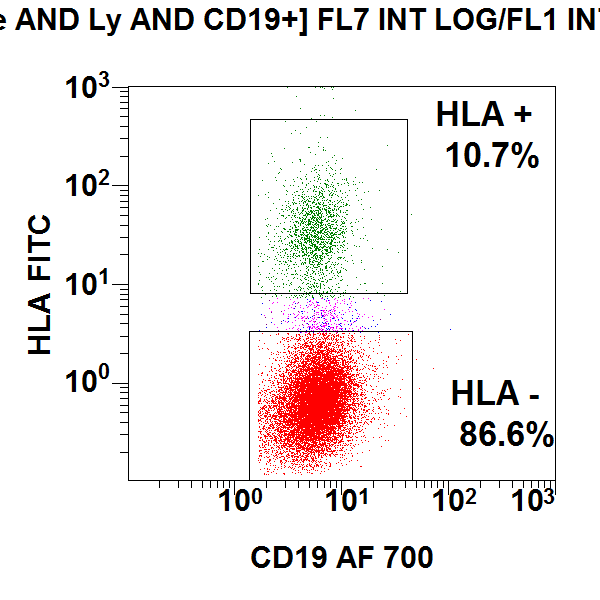


**HLA A32 pos Donor**

**HLA A32 neg Recipient**

**HLA A32**

**CD19**


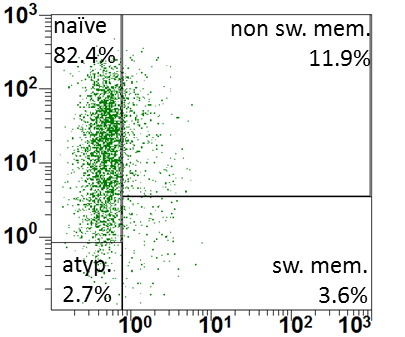

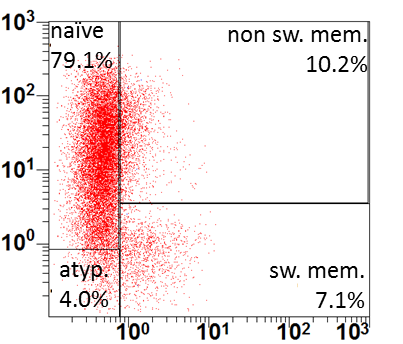

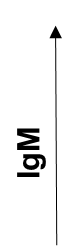

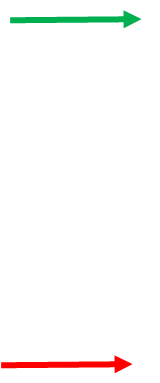


**IgD**

**CD27**

**Figure S3** In ADA deficiency (A. patient UPN 683) and in IL7R deficiency (B. patient UPN 569), autologous B cells show a normal differentiation indicating that the atypical distribution of B cell subpopulations described in Fig 1, 3, suppl. Fig 1 and suppl. Fig 2 are specific for patients with B-positive SCID caused by pathogenic variants in *IL2RG* or *JAK3*. Peripheral blood MNCs of the patients (A. 9 years post SCT, B. 12 years post SCT) were stained with donor specific anti-HLA- A2 (A. patient UPN 683) or anti-HLA-A32 (B. patient UPN 569), followed by staining with anti-CD19, anti-CD27, anti-IgM and anti IgD. Donor B cells (green: CD19+ and donor HLA pos.) **and** recipient B cells (red: CD19+ and donor HLA neg.) are equally able to switch to IgM-CD27+CD19+ B cells and show a considerable proportion of IgD-negative B cells.

**Supplemental Figure S4: Switched memory B cells in relation of the donor B cell proportion**

**A. B.**

reference values

| **Spearman r** |  |
| --- | --- |
| r | -0,6783 |
| 95% confidence interval | -0,9049 to -0,1522 |
|  |  |
|  |  |
| **P value** |  |
| P (two-tailed) | 0,0185 |
| P value summary | * |
| Exact or approximate P value? | Exact |
| Significant? (alpha = 0.05) | Yes |
|  |  |
|  |  |
| **Number of XY Pairs** | 12 |

**Figure S4 A.** Patients with low proportions (<20%, dotted line) of donor B cells show high percentages of switched memory B cells in their donor B cell population. Donor B cells of 12 patients (s. Fig 3) with IL2RG deficiency after haploidentical stem cell transplantation were detected by HLA-antibodies. The proportion of switched memory B cells (CD27+IgM-) within the donor B cell population (y-axis) is plotted against the proportion of donor B cells of CD19+ B cells (x-axis). Error bars indicate age-related reference values. Statistical analysis (Spearman r and P value) was performed by using GraphPad Prism 9.1.2. **B.** Longitudinal B cell chimerism analysis (donor cells of CD19+ B cells) with increasing proportions of switched memory B cells within the donor B cell population of patient UPN856 while donor-B cells declined (26% one year post SCT to 8.9% 10y post SCT). Age-related reference values are indicated as grey area.

**
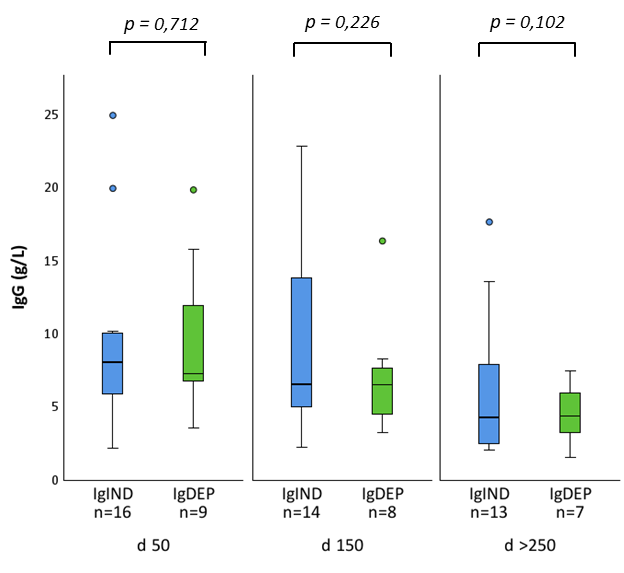
Supplemental Figure S5: Immunoglobulin levels in patients after hematopoietic stem cell transplantation (HSCT)**

**A.**

**
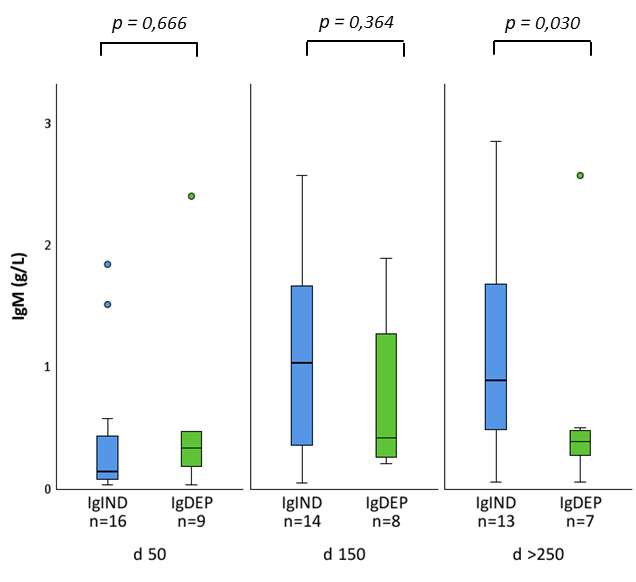
B.**

**Figure S5 A.** IgG and IgM-Levels in patients (cohort A) at day 50, 150 and more than 250 days after hematopoietic stem cell Transplantation (HSCT). There is no significant difference in the serum IgG-levels after HSCT in patients independent (IgIND, blue colour) or dependent (IgDEP, green colour) of immunoglobulin substitution. Because of the substitution of IgG it is not possible to distinguish between self-produced and substituted immoglobulin. **B.** For serum-IgM, a statistically significant difference between IgIND and IgDEP patients could be observered at more than 250 days after HSCT. Boxes represent interquartile ranges (25.-75. percentile), lines within the boxes indicate median values while the whiskers extend to 1,5 times the interquartile range with individual points for outliers.

**Supplemental Figure S6:**  **Definition of cut off by ROC-analysis**

| **​** | **​** | **CD27+IgM-/CD27+​** | | **​** |
| --- | --- | --- | --- | --- |
| ​ | ​ | < 4.7 %​ | > 4.7 %​ | ​ |
| **Need of Immuno-globulin** | IgIND​ | 1​ | 15 | 16 |
|  | IgDEP​ | 8 | 1​ | 9​ |
| ​ | ​ | 9 | 16 | ​ |


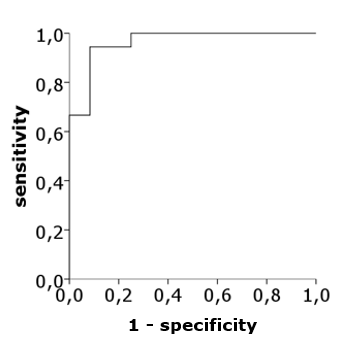


**Figure S6.** A ROC-analysis was used to determine the optimum cut-off value between the IgIND and IgDEP groups of cohort A. A percentage of more than 4.7% class-switched memory B cells within the CD27+ population with a sensitivity of 93,8% and a specificity of 89,9% was found to be the optimal cut-off value (by maximizing the Youden index) in our cohort.
